# Gene network reconstruction from microarray data

**DOI:** 10.1186/1753-6561-3-S4-S12

**Published:** 2009-07-16

**Authors:** Florence Jaffrezic, Gwenola Tosser-Klopp

**Affiliations:** 1 INRA AgroParisTech, Animal Genetics and Integrative Biology, Populations Statistics Genomes, 78350 Jouy-en-Josas, France; 2 Laboratoire de Génétique Cellulaire, INRA, UMR444, F-31326 Castanet-Tolosan, France

## Abstract

**Background:**

Often, software available for biological pathways reconstruction rely on literature search to find links between genes. The aim of this study is to reconstruct gene networks from microarray data, using Graphical Gaussian models.

**Results:**

The *GeneNet *R package was applied to the Eadgene chicken infection data set. No significant edges were found for the list of differentially expressed genes between conditions MM8 and MA8. On the other hand, a large number of significant edges were found among 85 differentially expressed genes between conditions MM8 and MM24.

**Conclusion:**

Many edges were inferred from the microarray data. Most of them could, however, not be validated using other pathway reconstruction software. This was partly due to the fact that a quite large proportion of the differentially expressed genes were not annotated. Further biological validation is therefore needed for these networks, using for example in vitro invalidation of genes.

## Introduction

Two main approaches have been proposed in the literature for gene network reconstruction from microarray data, namely Bayesian networks and Graphical Gaussian models. Bayesian networks are directed acyclic graphs, i.e. no feedback loop is possible. They are usually very computationally intensive and, as far as we are aware of, no R package is available for large-scale gene network reconstruction using Bayesian networks. On the other hand, Graphical Gaussian models are undirected graphs and are very computationally efficient. An R package is available for gene network reconstruction from microarray data using Graphical Gaussian models, namely *GeneNet *[1].

Werhli et al. [2] presented a comparison study between Bayesian networks and Graphical Gaussian models for gene network reconstruction. They concluded that both methods provided quite similar results for network reconstruction based on observed microarray data. We therefore chose, in this study, to base inference on Graphical Gaussian models.

### Graphical Gaussian models

Let ***X ***be the observed data matrix with *N *rows, corresponding to the number of samples, and *G *columns, corresponding to the number of genes. ***X ***is supposed to follow a multivariate normal distribution (***μ***, **Σ**), with mean vector ***μ ***= (*μ*_1_,...., *μ*_*G*_)*' *and positive-definite covariance matrix **Σ **= (*σ*_*ij*_)_(1≤*i*,*j*≤*G*)_.

Covariance parameters *σ*_*ij *_can also be written as: *σ*_*ij *_= *ρ*_*ij*_*σ*_*i*_*σ*_*j*_, where  and  are the variance terms for genes *i *and *j*, respectively. Parameter *ρ*_*ij *_corresponds to the Pearson correlation coefficient between genes *i *and *j*.

Let ***P ***be the Pearson correlation matrix: ***P ***= (*ρ*_*ij*_)_(1≤*i*,*j*≤*G*)_. A high correlation coefficient between two genes may indicate either [3]: *i*) a direct interaction between genes *i *and *j*; ii) an indirect interaction between these two genes; iii) a regulation of the two genes by a common gene. For network reconstruction we are only interested in direct interactions, represented by the partial correlation matrix **Π **= (*π*_*ij*_)_(1≤*i*,*j*≤*G*)_. Coefficient *π*_*ij *_represents the correlation between two genes *i *and *j *conditionally on all the other genes. It can be shown [3] that partial correlation matrix **Π **is related to the inverse of the covariance matrix **Σ **as follows:

(1)

with **Σ**^-1 ^= (*ω*_*ij*_), for 1 ≤ *i*, *j *≤ *G*.

Several steps are required for the construction of a Graphical Gaussian model network. First, the empirical covariance matrix has to be estimated:



Second, the partial correlation matrix has to be calculated using the previous equations. Finally, statistical tests can be performed to determine the partial correlation coefficients that are different from 0, and which correspond to the significant edges of the graph.

The procedure described above is, however, only applicable when sample size *N *is larger than the number of variables *G*. In fact, the sample covariance matrix is otherwise not positive-definite and cannot be inverted, which prevents a direct computation of the partial correlation matrix. In microarray experiments, however, we are very often in situations where sample size *N *is much smaller than the total number of genes *G*.

Schäfer and Strimmer [1] therefore proposed to use a shrunk estimate of the covariance matrix using a James-Stein estimator. The aim of this approach is to construct a well conditioned positive-definite matrix so that the matrix has full rank and can easily be inverted.

Let *λ *be a shrinkage coefficient (*λ *∈ [0, 1]). The shrunk covariance matrix Σ* is obtained as:

(2)

where  is the estimated empirical covariance matrix. Shrinkage parameter *λ *is chosen to minimize the mean-squared error (MSE) and can be determined analytically [1].

There are several possibilities for the choice of matrix ***T***. Schäfer and Strimmer [1] recommend for gene network reconstruction to shrink the correlation terms towards zero and to leave the diagonal terms as estimated by the empirical variances. In this case, shrinkage parameter *λ *can be estimated analytically as:

(3)

where *s*_*ij *_are the empirical covariance parameters.

An edge-specific local FDR procedure was then defined, based on the estimated partial correlation coefficients. As recommended by Efron [4], an edge is considered significant if its local FDR value is smaller than 20%.

### Application

The *GeneNet *R package [1] was applied to the Eadgene chicken infection data set [5] and the R code used to produce these analyses is available from the first author. We considered here the lists of differentially expressed genes obtained for two sets of conditions. In condition MA, chickens were infected at two weeks of age with a parasite called Eimeria maxima and two weeks later with the parasite called Eimeria acervulina, and in condition MM, chickens were infected first with E. maxima and afterwards with the same parasite E. maxima. Two time points were sampled post infection: 8 hours and 24 hours. At a 5% Benjamini-Hochberg (BH) threshold, 85 genes were found differentially expressed between groups MM and MA at 8 hours post infection, whereas 800 genes were found differentially expressed at a 5% BH threshold for condition MM between the two time points 8 and 24 hours. Due to the quite small number of biological replicates per condition (5 animals), network inference can only be performed on a few dozens of genes. For conditions MM8 and MM24, we therefore considered a more stringent BH threshold, with 116 differentially expressed genes at a 1% Benjamini-Hochberg threshold.

As no missing values are allowed in *GeneNet*, 58 genes were used for network reconstruction among the list of differentially expressed genes between conditions MM8 and MA8. Network inference was performed using the expression values for each condition independently. For this first analysis, no significant edges were found at the recommended 20% local FDR [4] threshold, for either condition MM8 or MA8.

For conditions MM8 and MM24, as no missing values are allowed, network reconstruction was based on 85 genes among the 116 found differentially expressed at a 1% Benjamini-Hochberg threshold.

For expression values observed in condition MM8, 2356 edges were found significant at the 20% local FDR threshold among the 85 genes, and even 1964 edges were found significant at the more stringent 5% local FDR threshold. Similarly, a very large number of edges were found significant between these 85 genes for condition MM24. In fact, 1760 edges were found significant at the 20% local FDR threshold, and 1156 at the 5% local FDR threshold.

**Figure 1:**
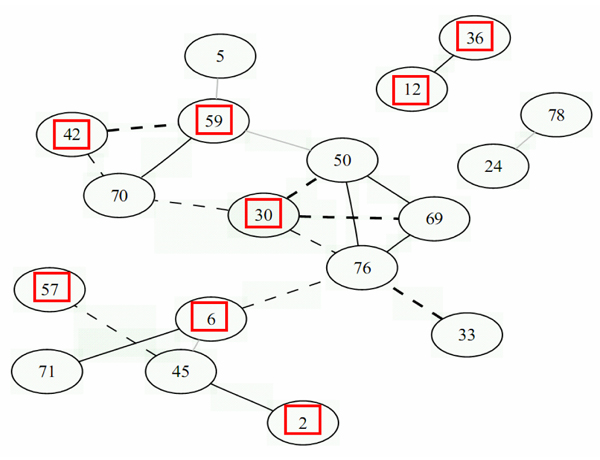
**Gene network for condition MM8**. Gene network for condition MM8 obtained with the *GeneNet *R package, for the 20 most significant edges. Solid lines represent positive relationships, dotted lines are negative relationships. The line intensities represent the strength of the relationships. Bold lines are stronger and light grey lines are weaker. Red squares represent annotated genes.

**Figure 2:**
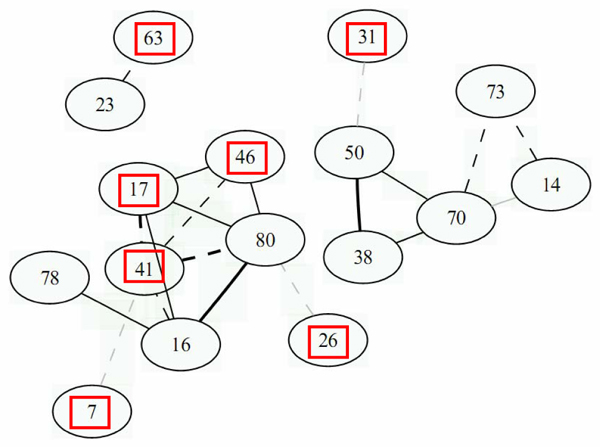
**Gene network for condition MM24**. Gene network for condition MM24 obtained with the *GeneNet *R package, for the 20 most significant edges. Legend for this graph is the same as for Figure 1.

Figures 1 and 2 show the graphs of the 20 most significant edges for both conditions, and Tables 1 and 2 provide the correspondence between the gene numbers given in the figures and their RIGG names and Human orthologs. It can be seen that there is very little overlapping between the two networks. In fact, only three genes were found in common in both graphs, and no link was conserved between both graphs. Furthermore, for condition MM8, among the 18 genes present in the graph, only 8 were annotated and for condition MM24, only 7 genes were annotated, which made it very difficult to validate the links found here using literature based pathway reconstruction software. Among the annotated genes present in these graphs, no links were found between them using either Ingenuity or Pathway Studio. Further biological validation is therefore required for this experiment using, for example, in vitro invalidation of genes, in order to confirm the links inferred here based on the gene expression measurements.

**Table 1:** Names of the annotated genes for condition MM8.

Gene number	RIGG name	Human ortholog HGNC
2	RIGG00270	C9orf80
6	RIGG01146	C6orf106
12	RIGG02317	SLC30A6
30	RIGG07776	SH2B2
36	RIGG09586	EFR3B
42	RIGG11710	SGK2
57	RIGG15302	GOLGB1
59	RIGG15630	PPP1R12A

**Table 2:** Names of the annotated genes for condition MM24.

Gene number	RIGG name	Human ortholog HGNC
7	RIGG01243	BAZ1B
17	RIGG03141	AP000775.4
26	RIGG07311	PRKCQ
31	RIGG08254	ABHD6
41	RIGG11399	C15orf27
46	RIGG12572	CCNY
63	RIGG15936	DTX4

## Discussion

Gene network reconstruction was based here on the expression data from this experiment only. It would be interesting to integrate some prior biological knowledge such as gene relationships already found in the literature, or to combine expression values from several studies, to have more power and accuracy for the edge detection.

Biological validation of the edges inferred here with Graphical Gaussian models was very difficult, mainly due to the lack of annotation for the lists of differentially expressed genes. An important effort therefore has to be made in the near future to obtain a more complete annotation of the chicken genome and other livestock species.

In the approach presented here, and based on partial correlations, it is only possible to model linear dependencies between genes. In order to take into account non linear relationships it may be possible, as suggested by Hausser and Strimmer [6] to use entropy instead of partial correlations to infer the edges between genes.

As only two time points were available in this study, static networks were considered here using the expression values at each time point separately. Several methods have recently been proposed for gene network reconstruction in time course studies, mainly based on VAR1 models [7]. If additional time points were added in the future to this experiment, it would be interesting to use these methods to study the gene relationships over time.

## Competing interests

The authors declare that they have no competing interests.

## Authors' contributions

F. Jaffrezic was in charge of the statistical analysis of the data and G. Tosser-Klopp of the biological interpretation of the results.
